# Physical activity and lung function association in a healthy community-dwelling European population

**DOI:** 10.1186/s12890-024-02979-x

**Published:** 2024-04-08

**Authors:** Sybile Collaud, Brice Touilloux, Christophe von Garnier, Pedro Marques-Vidal, Vanessa Kraege

**Affiliations:** 1 Department of Medicine, Internal Medicine, Lausanne University Hospital and University of Lausanne, Lausanne, Switzerland; 2 Division of Pulmonology, Department of Medicine and Specialties, Fribourg Hospital and University of Fribourg, Fribourg, Switzerland; 3 Division of Pulmonology, Department of Medicine, Lausanne University Hospital and University of Lausanne, Lausanne, Switzerland; 4 Medical Directorate, Lausanne University Hospital and University of Lausanne, Lausanne, Switzerland; 5 Innovation and Clinical Research Directorate, Lausanne University Hospital and University of Lausanne, Lausanne, Switzerland

**Keywords:** Accelerometry, Epidemiology, Exercise, Independent living, Respiratory function tests, Spirometry

## Abstract

**Background:**

The association of physical activity (PA) and lung function (LF) varies from no measurable effect to delayed LF decline. We assessed the association between accelerometery-assessed PA and LF in a sample of apparently healthy, community-dwelling subjects.

**Methods:**

We included two cross-sectional studies using data from the PneumoLaus study (2014–17 and 2018–21), conducted in Lausanne, Switzerland. PA was assessed by accelerometry and categorised as inactivity, light, moderate or vigorous. Forced expiratory volume in 1 second (FEV_1_), forced volume capacity (FVC) and maximal mid-expiratory flow (MMEF) were measured by spirometry and expressed in percentage of predicted value (PV).

**Results:**

Overall, 1′910 (54.7% women, 62.0 ± 9.7 years) and 1′174 (53.4% women, 65.8 ± 9.5 years) participants were included in the first and the second surveys, respectively. In both surveys, moderate and vigorous PA showed a weak but significant correlation with FEV_1_ in percentage (PV) (R = 0.106 and 0.132 for the first and 0.111 and 0.125 for the second surveys, *p* < 0.001). Similar correlations with FVC (*p* < 0.001) were found. Associations held irrespective of smoking status and remained after multivariable adjustment. Fewer associations were detected between LF and light PA or between MMEF and PA.

**Conclusion:**

Moderate and vigorous intensity PA are associated with increased LF regardless of smoking status in apparently healthy community-dwelling European population. These associations are statistically but not clinically significant due to the small correlation coefficients (R < 0.30), corresponding to a weak association.

**Supplementary Information:**

The online version contains supplementary material available at 10.1186/s12890-024-02979-x.

## Introduction

Physical activity (PA) has been shown to improve a wide range of physiological parameters, including lung function (LF) [1]. A prospective study over 25 years, published in 2003, reported a positive association between PA levels and forced expiratory volume in 1 second (FEV_1_) and forced vital capacity (FVC) [2]. The authors concluded that PA may delay the decline in LF that occurs in middle- and old-age, regardless of smoking status.

However, other cross-sectional and prospective studies only found this association among smokers [3, 4] and another found no association between PA and spirometric indices [5]. These divergent results may be due to the difficulty of PA quantification. Indeed, some studies used PA questionnaires [2–4, 6–9] which can be biased by subjective input. A study conducted in former and current smokers assessed PA via accelerometery and reported a positive relationship between moderate-to-vigorous PA (MVPA) and LF [10]. Consequently, there is conflicting or minor evidence on the association between objectively assessed PA and LF, and the degree of PA necessary to maintain adequate LF.

We aimed to repeat the associations between objectively assessed PA and LF in a sample of healthy, community-dwelling subjects in Switzerland, overall and according to smoking status, and to study this association at two different time points of our cohort. Our hypothesis was that these would show a good to strong correlation.

## Methods

### PneumoLaus study

The PneumoLaus study is part of the CoLaus|PsyCoLaus study (https://www.colaus-psycolaus.ch), an ongoing prospective study aiming to assess the determinants of cardiovascular and psychiatric diseases using a population-based sample drawn from the city of Lausanne, Switzerland [11]. In June 2014, all participants of the CoLaus|PsyCoLaus study were invited to take part in the PneumoLaus study. Baseline examinations were conducted between June 2014 and August 2017, and the follow-up survey was conducted between June 2018 and February 2021. All ethnic groups were included as the general population was randomly invited to participate in the CoLaus|PsyCoLaus study.

### Ethical statement

The CoLaus|PsyCoLaus studiy were approved by the local Ethics Committee and participants provided written informed consent (https://www.cer-vd.ch; project number PB_2018–00038, reference 239/09).

### Spirometry

PneumoLaus methodology was already described [12]. Briefly, LF was assessed using a MasterScreen-PFT spirometer (Carefusion, Hoechberg, Germany), with Sentry Suite software (Version 2.17). Each manoeuvre was automatically assessed by computer, based upon acceptability and reproducibility criteria according to the 2005 American Thoracic Society– European Respiratory Society standards [13].

Reference values were applied according to the Global Lung Function Initiative (GLI) 2012, adjusting for the following ethnic origins: Caucasian, African, Northeast Asian, Southeast Asian and other [14]. If FEV_1_/FVC or FVC was found to be below the lower limits of normal (LLN), spirometry was repeated 10–15 minutes after administration of 4 × 100 μg of salbutamol via a metered-dose inhaler and a spacer. Normal spirometry was defined by baseline FEV_1_/FVC ratio and FVC above LLN, representing the lower 5th percentile (corresponding to a z-score of − 1.645) based on age, sex, height and ethnicity [15]. The maximal mid expiratory flow (MMEF) was defined by the mean forced expiratory flow between 25 and 75% of the FVC. An experienced respiratory technician and a consultant pulmonologist evaluated the quality of spirometry manoeuvres. Spirometry was included using recognised acceptability and reproducibility criteria [16].

### Physical activity assessment

PA was assessed using a wrist-worn triaxial accelerometer (*GENEActiv*, Activinsights Ltd., United Kingdom, https://www.activinsights.com). The accelerometer has been validated against caliometry demonstrating excellent correlations against METs (Metabolic Equivalent of Task). The area under the Receiver Operating Characteristic (ROC) curves to discriminate sedentary activity ranged from 0.844–0.896, and for moderate to vigorous activity from 0.991–0.993 [17].

The devices were pre-programmed with a 50 Hz sampling frequency and subsequently attached to the participants’ right wrist. Participants were requested to wear the device continuously for 14 days in their free-living conditions. A valid day was defined as ≥10 hours of diurnal wear-time on weekdays and ≥ 8 hours on week-end days. At least 5 week days and 2 week-end days of valid data were required [18, 19].

PA was categorised into inactivity (< 85 m-g), light (85–180 m-g), moderate (181–437 m-g) and vigorous (> 437 m-g) PA, based on the thresholds defined by White et al. [20] and the average daily time spent within each category was utilised for analysis. MVPA was obtained by summing time spent in moderate and vigorous PA.

### Other covariates

Educational level was categorised into high (university), middle (high school), and low (apprenticeship or mandatory) [21]. Nationality was categorised as born in Switzerland or not. Marital status was categorised as living with or without a partner. Smoking status was self-reported and categorised as never, former, or current smoker [22].

Body weight and height were measured with participants barefoot and in light indoor clothes. Body weight was measured in kilograms to the nearest 100 g using a Seca® scale (Hamburg, Germany). Height was measured to the nearest 5 mm using a Seca® (Hamburg, Germany) height gauge. Body mass index (BMI) was categorised as normal (< 25 kg/m^2^), overweight (≥25 and < 30 kg/m^2^) and obese (≥30 kg/m^2^) [23].

### Inclusion and exclusion criteria

Participants from the PneumoLaus study (2014–17 and 2018–21) were considered as eligible. Those with possible restrictive ventilatory impairment defined as FVC < LLN before and after bronchodilation or obstructive ventilatory impairment defined as FEV_1_/FVC < LLN or missing any information concerning PA and smoking status were excluded.

## Data analysis

Data was analysed employing Stata version 17.0 for Windows (Stata Corp, College Station, Texas, USA). Descriptive statistics were presented as number of participants (percentage) for categorical variables or as average ± standard deviation for continuous variables. Between-group comparisons were performed using chi-square or Fisher’s exact test for categorical variables and student’s t-test or analysis of variance for continuous variables.

Within each PA level, the associations between LF (in absolute volumes and in percentage of predicted value) and time spent in the corresponding PA level were assessed by Spearman rank correlation and the coefficients of determination (R-squared or R^2^), defined as the square value of the correlation coefficient or the percentage of variance explained by the multivariable linear regression model were computed. The R^2^ coefficient was considered “high” when it was > 0.70, “good” between 0.50–0.70, “fair” between 0.30–0.50 and “weak or no association” if it was < 0.30 [24]. Multivariable analysis was conducted using linear regression, with LF as the dependent variable and time spent in a PA level as independent variable. Adjustments were performed for age (continuous), BMI (continuous) and smoking category (never, former, current) and results were expressed as standardised beta coefficients. Statistical significance was considered for a two-sided test with *p* < 0.05. A mixed method analysis was then performed, grouping the data from both follow-ups, thus including some participants with data in each.

A final analysis categorised the duration of MVPA in tertiles and compared LF parameters between them.

## Results

### Selected participants

**Fig. 1:**
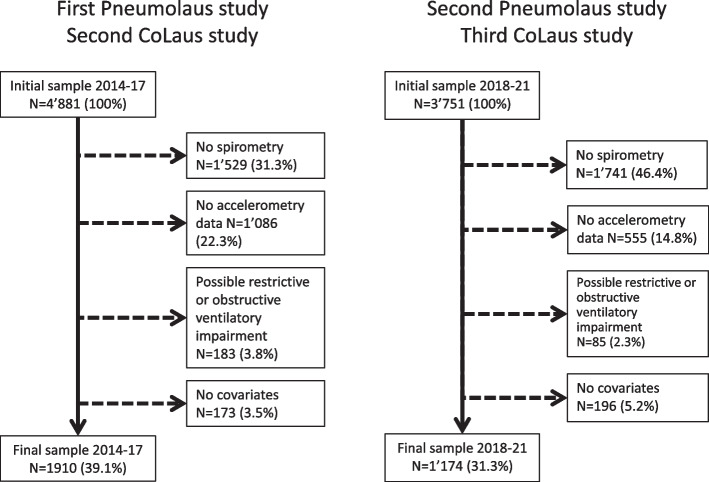
Selection procedure for the participants of the first and second PneumoLaus studies, Lausanne, Switzerland

Of the initial 4881 participants of the baseline survey, 1910 (39.1% participation rate, 54.7% women, 62.0 ± 9.7 years) were included in the analysis. Of the initial 3751 participants of the follow-up survey, 1174 (participation rate 31.3, 53.4% women, 65.8 ± 9.5 years) were included. The reasons for exclusion and the characteristics of the included and excluded participants are summarised in Fig. 1 and Supplementary Table 1, respectively. In the baseline survey, included participants were younger, more frequently professionally active, of higher educational level and less frequently smokers or hypertensive than excluded participants. In the follow-up survey, included participants were older, less professionally active, more frequently of middle educational level, and less frequently smokers than excluded participants.

### Association between PA and spirometry metrics as % of predicted values

The results of bivariate and multivariable analyses for percentage of PV are summarised respectively in Tables 1 and 2, and the R^2^ for bivariate associations are provided in Supplementary Table 2. In both studies and analyses, vigorous PA was significantly and positively associated with FEV_1_ and FVC except in current smokers of the follow-up study. R^2^ were low, ranging between 0 and 4.2%.

**Table 1:** Spearman non-parametric correlation coefficients between physical activity levels and spirometry results in percentage of predicted value, overall and stratified by smoking status, Pneumolaus baseline (2014–2017) and follow-up (2018–2021), Lausanne, Switzerland

	All participants			Never or former smokers			Current smokers		
	FEV_1_% PV	FVC % PV	MMEF % PV	FEV_1_% PV	FVC % PV	MMEF % PV	FEV_1_% PV	FVC % PV	MMEF % PV
**Baseline**	***N*** **= 1910**			***N*** **= 1604**			***N*** **= 306**		
Inactivity	**−0.106**	**−0.113**	− 0.039	**−0.108**	**− 0.123**	−0.034	− 0.072	−0.055	− 0.021
Light PA	**0.101**	**0.108**	0.032	**0.085**	**0.097**	0.013	**0.157**	**0.159**	0.075
Moderate PA	**0.106**	**0.118**	0.033	**0.078**	**0.098**	0.006	**0.193**	**0.195**	0.097
Vigorous PA	**0.132**	**0.137**	**0.059**	**0.109**	**0.123**	0.031	**0.206**	**0.192**	**0.135**
**Follow-up**	***N*** **= 1174**			***N*** **= 1022**			***N*** **= 152**		
Inactivity	**−0.057**	**−0.063**	−0.013	**−0.065**	**−0.074**	−0.018	0.010	0.021	0.028
Light PA	**0.101**	**0.134**	−0.007	**0.111**	**0.142**	0.002	0.052	0.087	−0.064
Moderate PA	**0.111**	**0.143**	−0.001	**0.124**	**0.157**	0.003	0.030	0.047	−0.034
Vigorous PA	**0.125**	**0.159**	0.017	**0.137**	**0.179**	0.011	0.038	0.004	0.037

*FEV*_*1*_ forced expired volume in 1 second, *FVC* forced vital capacity, *MMEF* maximal mid-expiratory flow, *PA* physical activity, *PV* predicted value. Results are expressed as Spearman rank correlation coefficients. Significant (*p* < 0.05) correlations are indicated in bold characters

**Table 2:** Multivariable analysis of the associations between physical activity levels and spirometry results in percentage of predicted value, overall and stratified by smoking status, Pneumolaus baseline (2014–2017) and follow-up (2018–2021), Lausanne, Switzerland

	All participants			Never or former smokers			Current smokers		
	FEV_1_% PV	FVC % PV	MMEF % PV	FEV_1_% PV	FVC % PV	MMEF % PV	FEV_1_% PV	FVC % PV	MMEF % PV
**Baseline**	***N*** **= 1910**			***N*** **= 1604**			***N*** **= 306**		
Inactivity	**−0.094**	**−0.098**	−0.033	**−0.100**	**−0.108**	− 0.035	−0.065	− 0.049	−0.026
Light PA	**0.087**	**0.080**	0.029	**0.072**	**0.070**	0.016	**0.170**	**0.143**	0.107
Moderate PA	**0.080**	**0.073**	0.023	**0.060**	**0.058**	0.008	**0.208**	**0.179**	**0.122**
Vigorous PA	**0.079**	**0.072**	0.037	**0.068**	**0.067**	0.020	**0.182**	**0.125**	**0.200**
**Follow-up**	***N*** **= 1174**			***N*** **= 1022**			***N*** **= 152**		
Inactivity	−0.046	−0.035	−0.018	−0.056	−0.045	− 0.029	0.014	0.032	0.034
Light PA	**0.094**	**0.110**	0.007	**0.106**	**0.121**	0.004	0.019	0.035	−0.068
Moderate PA	**0.089**	**0.100**	0.003	**0.097**	**0.111**	−0.002	0.036	0.034	−0.024
Vigorous PA	**0.086**	**0.071**	0.052	**0.102**	**0.096**	0.046	−0.007	−0.059	0.078

*FEV*_*1*_ forced expired volume in 1 second, *FVC* forced vital capacity, *MMEF* maximal mid-expiratory flow, *PA* physical activity, *PV* predicted value. Results are expressed as beta-standardized coefficients. Statistical analysis conducted using multivariable regression adjusting for body mass index (continuous), and for smoking categories (never, former, current) when all participants were considered. Significant (*p* < 0.05) associations are indicated in bold characters

In the multivariable analysis of both studies, moderate PA was significantly and positively associated with FEV_1_ and FVC except in current smokers of the follow-up. No significant association was found between any PA levels and MMEF, except for a positive association for moderate and vigorous PA in current smokers of the baseline study. Significant associations were found between light PA and both FVC and FEV_1_ as % of PV, except for current smokers of the follow-up.

Table 3 shows the mixed method analysis, merging both follow-ups, and including repeated measures of participants who performed LF in both follow-ups. The results confirm those obtained separately for each follow-up. For example, an increase of 15 minutes in inactivity leads to a decrease in FEV_1_ of 0.12%.

**Table 3:** Multivariable analysis of the associations between physical activity levels and spirometry results in percentage of predicted value, regression coefficients expressed for a percent-change per 15-minute period of PA, overall and stratified by smoking status, Pneumolaus baseline (2014–2017) and follow-up (2018–2021), Lausanne, Switzerland

	All participants			Never or former smokers			Current smokers		
	FEV_1_% PV	FVC % PV	MMEF % PV	FEV_1_% PV	FVC % PV	MMEF % PV	FEV_1_% PV	FVC % PV	MMEF % PV
	***N*** **= 3084**			***N*** **= 2626**			***N*** **= 458**		
Inactivity	**−0.12** **(−0.21; −0.03)**	**− 0.13** **(− 0.22; − 0.04)**	−0.12 (− 0.34; 0.1)	**−0.10** **(− 0.20; − 0.01)**	**−0.13** **(− 0.22; − 0.03)**	−0.09 (− 0.33; 0.14)	**−0.29** **(− 0.55; − 0.02)**	−0.20 (− 0.43; 0.02)	−0.36 (−1.01; 0.29)
*P*-value	0.007	0.004	0.283	0.035	0.011	0.438	0.035	0.080	0.281
Light PA	**0.35** **(0.18; 0.52)**	**0.37** **(0.22; 0.52)**	0.13 (−0.27; 0.52)	**0.30** **(0.12; 0.48)**	**0.34** **(0.18; 0.5)**	0.01 (−0.42; 0.43)	**0.65** **(0.19; 1.12)**	**0.54** **(0.10; 0.98)**	0.93 (−0.10; 1.97)
*P*-value	< 0.001	< 0.001	0.528	0.001	< 0.001	0.978	0.006	0.015	0.077
Moderate PA	**0.76** **(0.37; 1.15)**	**0.81** **(0.44; 1.18)**	0 (−0.93; 0.93)	**0.58** **(0.18; 0.99)**	**0.70** **(0.31; 1.09)**	−0.44 (−1.42; 0.53)	**2.24** **(1.07; 3.42)**	**1.83** **(0.69; 2.97)**	**3.20** **(0.56; 5.84)**
*P*-value	< 0.001	< 0.001	0.999	0.005	< 0.001	0.374	< 0.001	0.002	0.018
Vigorous PA	1.05 (−0.19; 2.30)	**1.34** **(0.26; 2.42)**	−0.24 (−3.32; 2.84)	1.02 (− 0.19; 2.24)	**1.38** **(0.27; 2.49)**	− 0.80 (− 3.79; 2.19)	5.48 (− 0.50; 11.5)	3.28 (−2.82; 9.37)	**17.7** **(6.56; 28.9)**
*P*-value	0.097	0.015	0.879	0.099	0.015	0.600	0.073	0.292	0.002

*FEV*_*1*_ forced expired volume in 1 second, *FVC* forced vital capacity, *MMEF* maximal mid-expiratory flow, *PA* physical activity, *PV* predicted value. Results are expressed as regression coefficients for a percent change per 15-minute period of inactivity or PA and 95% confidence interval. Statistical analysis conducted using mixed models for repeated measures adjusting for body mass index (continuous), and for smoking categories (never, former, current) when all participants were considered. Significant (*p* < 0.05) associations are indicated in bold characters

Similar findings were found upon LF in absolute values (Supplementary Tables 3, 4 and 5). In fact, only association between vigorous PA and both FEV_1_ and FVC were significantly positive when adjusting for BMI and age. A visual depiction of the bivariate associations between spirometry values and vigorous PA, respectively inactivity, is provided in Supplementary Figs. 1 and 2.

## Discussion

For most PA parameters, we observed small correlations with LF parameters, with R^2^ below 10%. This suggests that LF has limited importance in explaining PA in a large, healthy, community-dwelling population, despite statistically significant associations even after adjusting for age and BMI.

The significantly positive correlation seen between vigorous PA and absolute volume persisted for FEV_1_ and FVC as % of PV, except for current smokers in the follow-up study, also due to a lack of power. There was no association between MMEF in % of PV and PA. Nevertheless, when analysed univariately with spirometry values as % of PV, the correlation with moderate PA remained.

The association between PA and lung volumes could be partly due to the influence of different lifestyles on lung volumes. Just as PA, certain diets, such as the Mediterranean one, have a positive association with LF as they reduce inflammation in tissues, including in lungs [25, 26]. A positive association has been demonstrated both in a population without lung disease [25] and in case of cystic disease or chronic obstructive pulmonary disease [27–29].

### Comparison with other studies

The present study is consistent with the positive association found in three other cross-sectional studies which also used accelerometer-assessed PA [10, 30, 31]. In contrast to our study, the association was described as stronger for former and current smokers compared to non-smokers [10, 30] but this difference might only be due to lack of power of our small smoker group. On the contrary, Barboza et al. [32], who also used accelerometer-assessed PA, found no association between PA and LF, nor did Smith et al. [5]. Our distinct results may be attributed to the difference in definition of PA intensity levels, as several studies assessed PA based on questionnaires and others on accelerometry but without forcibly applying the same thresholds as we did. Similarly, there were differences between the studied populations. Ours was older: mean 62.0 ± 9.7 years at baseline and 65.8 ± 9.5 years at follow-up versus 47 ± 14 years (males) and 53 ± 8 years (females) in Barboza et al. [32] and 15.2 ± 0.25 years in Smith et al. [5]. Our population was also less overweight: mean BMI 26.3 ± 4.6 kg/m^2^ at baseline and 26.2 ± 4.3 at follow-up versus 27.4 ± 4.5 kg/m^2^ (males) and 29.8 ± 7.07 kg/m^2^ (females) in Barboza et al. [32]. In fact, Barboza et al. [32] used both questionnaire and accelerometery-assessed PA but only show accelerometer-derived inactivity data. Also, the device was worn on the wrist in our study, versus the hip in the others [5, 32]. The waist location has indeed been shown to have better accuracy, followed by the left and then the right wrist, which was used in our study [33]. As inflammation increases with age, one hypothesis to explain our findings could be that PA counterbalances excessive inflammation, which is not present in youth [26]. Indeed, Garcia-Aymerich et al. found an association between PA and LF only in older people (> 40 years) [3], already starting with moderate PA. Our study seems to confirm the age-related association but not the association between moderate PA and spirometric indices. Moreover, as shown in Rose et al.s systematic review and metanalysis, the intensity of exercise did not influence chronic inflammatory response in general, even though sub-analyses suggest that higher training intensity may have more effect in middle-aged adults [34]. For ease of understanding, Table 4 summarises the results of the cited studies.

**Table 4:** Summary of cited studies

Authors	Year of publication	Design	Number of subjects	Measurement method		Association between physical activity and lung volumes
				Questionnaire	Accelerometer	
**Pelkonen et al.** [2]	2003	prospective	207	X		positive
**Garcia-Aymerich et al.** [3]	2007	prospective	6′790	X		positive
**Fuertes et al.** [4]	2018	prospective	3′912	X		positive association only in smokers
**Smith et al.** [5]	2016	cross-sectional	895	X	X	none
**Bédard et al.** [6]	2020	longitudinal	753	X		positive association only in smokers
**Svartengren et al.** [7]	2020	cross-sectional	22′743	X		positive
**Luzak et al.** [8]	2018	cross-sectional	1132	X		positive
**Nystad et al.** [9]	2006	prospective	8′047	X		positive
**Benadjaoud et al.** [10]	2019	cross-sectional	3′063		X	positive
**Luzak et al.** [30]	2017	cross-sectional	1′132		X	positive
**Schweitzer et al.** [31]	2017	cross-sectional	40		X	positive
**Barboza et al.** [32]	2016	cross-sectional	62	X	X	none

### Strengths and limitations

The strength of this study is the objectively assessed PA by accelerometery and LF by spirometry. As this study was conducted in a sample of apparently healthy, community-dwelling people, our results indicate that maintaining an adequate level of PA is associated with a better LF. Finally, our sample size is larger than most other studies conducted in healthy people (*n* = 62 for Barboza et al. [32], *n* = 322 for Luzak et al. [30], *n* = 895 for Smith et al. [5]), thus allowing a bigger statistical power.

Our study also has limitations. First, it was conducted in a single city with mainly subjects of European ancestry, hence, results might not be generalisable in other settings or other ethnicities, although we believe that such associations should hold in most cases. Second, the cross-sectional design does not allow to establish the cause-effect relationship between PA and LF or to assess whether a long-term relationship between PA and LF exists. A dedicated prospective analysis would be necessary to answer this. Third, we did not determine the correlation between PA and static lung volumes such as total lung capacity, functional residual capacity, and residual volume. Fourth, due to low R^2^, clinical significance is not guaranteed despite statistically significative results, as shown in other studies. A meta-analysis would allow to draw stronger conclusions. Nevertheless, this small fraction of influence on spirometry metrics is similar or even higher than that obtained in polygenic risk scores for asthma, where 95% of R^2^ values are below 6% [35] or in similar scores in psychiatry, where R^2^ reach 2% for major depressive disorders, 5% for bipolar disorders or 6% for attention deficit hyperactivity disorders [36–39]. Fifth, the accelerometer was positioned in the dominant (right) wrist, contrary to other studies that used the nondominant side. This was done to increase participation, as wearing the accelerometer on the left wrist would interfere with the wristwatch that most participants wore. A study [33] suggested that results obtained using the right wrist might be less reliable than using the left one, but the study was conducted during a very limited period of time (1 hour) and in a laboratory setting. Hence, the findings might not be applicable to free-living people. Nevertheless, it would be important that other studies be conducted using the accelerometer on the nondominant hand and with a sample size like ours.

## Conclusion

The present study shows a weak correlation between vigorous PA and MVPA, with better LF, irrespective of smoking status, in an apparently healthy community-dwelling population. Lesser degrees of PA have no significant impact on LF.

### Supplementary Information

**Supplementary Material 1.**

**Supplementary Material 2.**

**Supplementary Material 3.**

## Data Availability

The data of CoLaus|PsyCoLaus and PneumoLaus studies used in this article cannot be fully shared as they contain potentially sensitive personal information on participants. According to the Ethics Committee for Research of the Canton of Vaud, sharing these data would be a violation of the Swiss legislation with respect to privacy protection. However, coded individual-level data that do not allow researchers to identify participants are available upon request to researchers who meet the criteria for data sharing of the CoLaus|PsyCoLaus Datacenter (CHUV, Lausanne, Switzerland). Any researcher affiliated to a public or private research institution who complies with the CoLaus|PsyCoLaus standards can submit a research application to or research.psycolaus@chuv.ch. Proposals requiring baseline data only, will be evaluated by the baseline (local) Scientific Committee (SC) of the CoLaus and PsyCoLaus studies. Proposals requiring follow-up data will be evaluated by the follow-up (multicentric) SC of the CoLaus|PsyCoLaus cohort study. Detailed instructions for gaining access to the CoLaus|PsyCoLaus data used in this study are available at https://www.colaus-psycolaus.ch/professionals/how-to-collaborate/.

## Abbreviations

PA: Physical activity
LF: Lung functions
FEV_1_: Forced expiratory volume in 1 second
FVC: Forced vital capacity
MET: Metabolic equivalent of task
MMEF: Maximum mid-expiratory flow
MVPA: Moderate and vigorous physical activity
GLI: Global lung initiative
ROC: Receiver operating characteristic
