# Supplementary material for: Physical activity and lung function association in a healthy community-dwelling European population
Source: BMC Pulm Med. 2024 Apr 8;24:169. doi: 10.1186/s12890-024-02979-x (PMC11003054; doi:10.1186/s12890-024-02979-x)
Supplement: Supplementary file 1 — Supplementary Material 1. [file 12890_2024_2979_MOESM1_ESM.pdf]

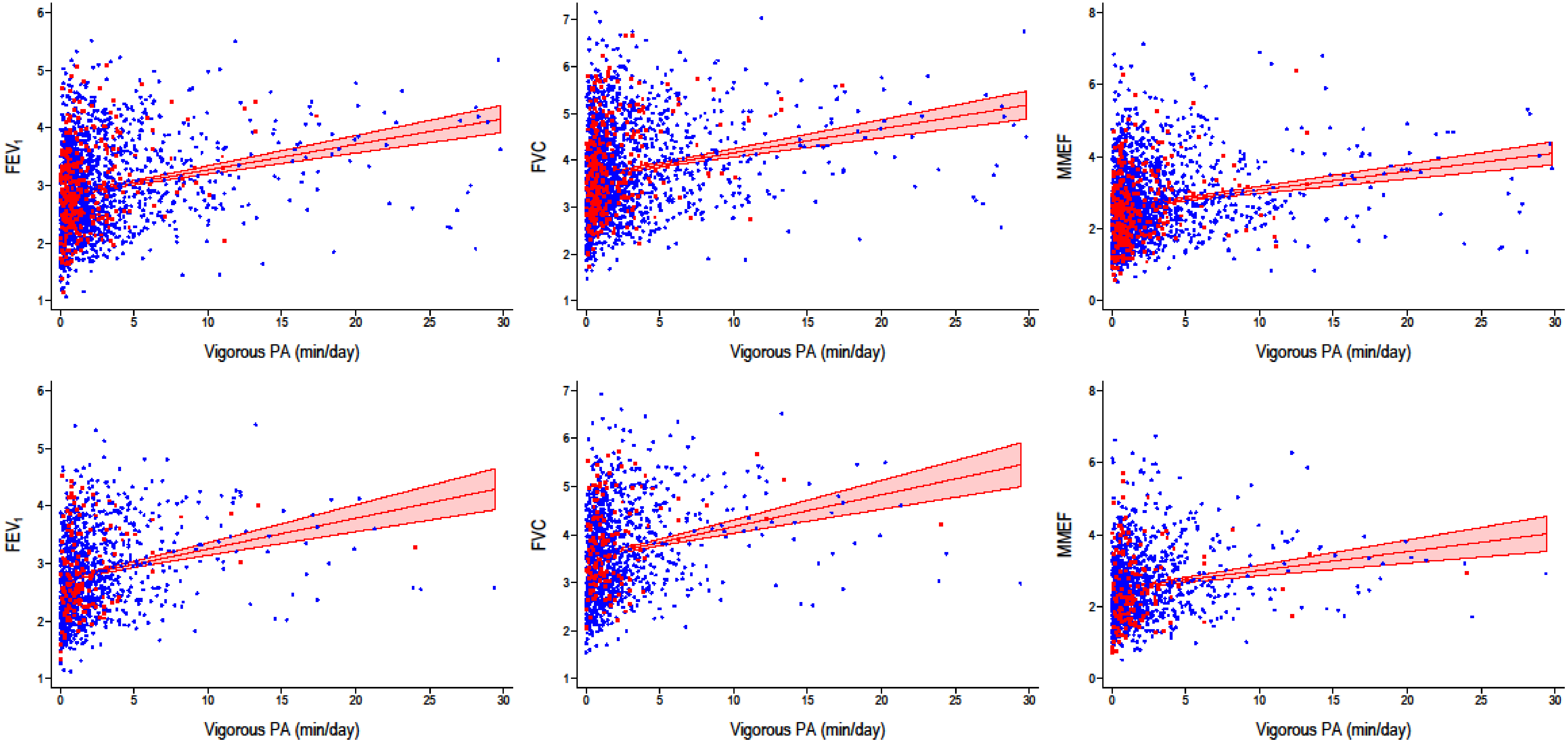


**Supplementary figure 1 :** Bivariate associations between vigorous physical activity (PA) and spirometry results in absolute volumes upon baseline study (three top graphs) and at follow-up (three bottom graphs). Blue dots represent never or former smokers; red dots represent current smokers. $FEV_1$ = Forced Expiratory Volume in 1 second ; FVC = Forced Vital Capacity ;  MMEF = Maximum Mid-Expiratory Flow.
