# Supplementary material for: Physical activity and lung function association in a healthy community-dwelling European population
Source: BMC Pulm Med. 2024 Apr 8;24:169. doi: 10.1186/s12890-024-02979-x (PMC11003054; doi:10.1186/s12890-024-02979-x)
Supplement: Supplementary file 2 — Supplementary Material 2. [file 12890_2024_2979_MOESM2_ESM.pdf]

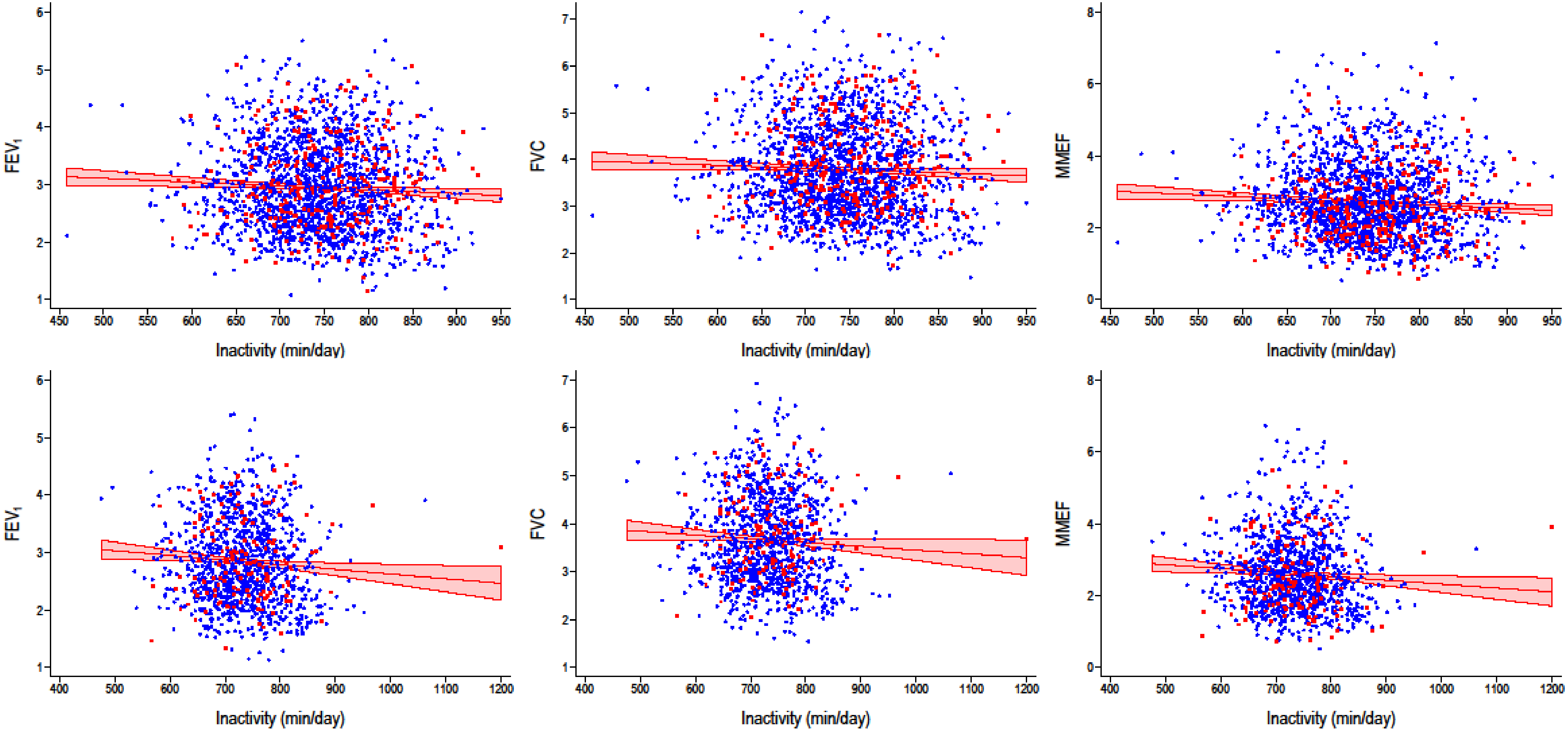

Supplementary figure 2 : Bivariate associations between inactivity and spirometry results in absolute volumes upon baseline study (three top graphs) and at follow-up (three bottom graphs). Blue dots represent never or former smokers; red dots represent current smokers. FEV_1 = Forced Expiratory Volume in 1 second ; FVC = Forced Vital Capacity ; MMEF = Maximum Mid-Expiratory Flow.
