# Supplementary material for: Physical activity and lung function association in a healthy community-dwelling European population
Source: BMC Pulm Med. 2024 Apr 8;24:169. doi: 10.1186/s12890-024-02979-x (PMC11003054; doi:10.1186/s12890-024-02979-x)
Supplement: Supplementary file 3 — Supplementary Material 3. [file 12890_2024_2979_MOESM3_ESM.pdf]

Supplementary information

Supplementary table 1: characteristics of included and excluded participants, Pneumolaus baseline and follow-up, Lausanne, Switzerland.

	Baseline (2014-2017)			Follow-up (2019-2021)		
	**Included**	**Excluded**	**P-value**	**Included**	**Excluded**	**P-value**
Sample size	1910	2971		1174	2577	
Women	1044 (54.7)	1645 (55.4)	0.627	627 (53.4)	1457 (56.5)	0.073
Age (years)	62.0 ± 9.7	63.5 ± 10.9	<0.001	65.8 ± 9.5	65.0 ± 10.1	0.019
Body mass index (kg/m^2)	26.3 ± 4.6	26.5 ± 4.8	0.099	26.2 ± 4.3	26.4 ± 4.8	0.243
Abdominal obesity (WHO)*	674 (35.3)	1019 (39.6)	0.003	447 (38.1)	903 (38.0)	0.968
Arterial hypertension §	795 (41.6)	1468 (52.9)	<0.001	581 (49.6)	1280 (52.0)	0.189
Professionally active	1093 (57.8)	1337 (49.6)	<0.001	524 (44.8)	1239 (48.4)	0.040
Education level			<0.001			0.037
High	424 (22.2)	607 (20.5)		260 (22.2)	586 (22.8)	
Middle	548 (28.7)	710 (23.9)		353 (30.1)	672 (26.1)	
Low	937 (49.1)	1651 (55.6)		561 (47.8)	1317 (51.2)	
Currently drinks alcohol	1562 (81.8)	1857 (75.9)	<0.001	958 (81.6)	1266 (78.1)	0.022
Smoking status			<0.001			0.002
Never	850 (44.5)	1036 (40.1)		540 (46.0)	955 (43.0)	
Former	754 (39.5)	1000 (38.7)		482 (41.1)	874 (39.4)	
Current	306 (16.0)	549 (21.2)		152 (13.0)	392 7.7)	

* World Health Organisation (WHO) § defined as blood pressure ≥140/90 mm Hg or antihypertensive drug treatment. Results are expressed as number of participants (column percentage) for categorical variables and as mean ± standard deviation for continuous variables. In excluded participants, percentages are based on non-missing data and might not add up to the total. Education level: low = base education and apprenticeship; middle = high school; high = university. Between-group comparisons performed using chi-square for categorical variables and student's t-test for continuous variables.

Supplementary table 2: Coefficients of determination between physical activity levels and spirometry results in percentage of predicted value, overall and stratified by smoking status, Pneumolaus baseline (2014-2017) and follow-up (2018-2021), Lausanne, Switzerland.

	All participants			Never or former smokers			Current smokers		
	**FEV_1 % PV**	**FVC % PV**	**MMEF % PV**	**FEV_1 % PV**	**FVC % PV**	**MMEF % PV**	**FEV_1 % PV**	**FVC % PV**	**MMEF % PV**
**Baseline**		**N=1910**			**N=1604**			**N=306**	
Inactivity	**1.1%**	**1.3%**	0.2%	**1.2%**	**1.5%**	0.1%	0.5%	0.3%	0.0%
Light PA	**1.0%**	**1.2%**	0.1%	**0.7%**	**0.9%**	0.0%	**2.5%**	**2.5%**	0.6%
Moderate PA	**1.1%**	**1.4%**	0.1%	**0.6%**	**1.0%**	0.0%	**3.7%**	**3.8%**	0.9%
Vigorous PA	**1.7%**	**1.9%**	**0.3%**	**1.2%**	**1.5%**	0.1%	**4.2%**	**3.7%**	**1.8%**
**Follow-up**		**N=1174**			**N=1022**			**N=152**	
Inactivity	**0.3%**	**0.4%**	0.0%	**0.4%**	**0.5%**	0.0%	0.0%	0.0%	0.1%
Light PA	**1.0%**	**1.8%**	0.0%	**1.2%**	**2.0%**	0.0%	0.3%	0.8%	0.4%
Moderate PA	**1.2%**	**2.0%**	0.0%	**1.5%**	**2.5%**	0.0%	0.1%	0.2%	**0.1%**
Vigorous PA	**1.6%**	**2.5%**	0.0%	**1.9%**	**3.2%**	0.0%	0.1%	0.0%	**0.1%**

FEV_1 = forced expired volume in one second; FVC = forced vital capacity; MMEF = maximum mid-expiratory flow; PA = physical activity; PV = predicted value. Results are expressed as percentage. Significant ($p<0.05$) associations are indicated in bold characters.

Supplementary table 3: Spearman non-parametric correlation coefficients between physical activity levels and spirometry results in absolute volumes, overall and stratified by smoking status, Pneumolaus baseline (2014-2017) and follow-up (2018-2021), Lausanne, Switzerland.

	All participants			Never or former smokers			Currents smokers		
	**FEV_1**	**FVC**	**MMEF**	**FEV_1**	**FVC**	**MMEF**	**FEV_1**	**FVC**	**MMEF**
**Baseline**		**N=1910**			**N=1604**			**N=306**	
Inactivity	**-0.053**	-0.041	**-0.070**	**-0.066**	**-0.057**	**-0.076**	0.006	0.020	-0.018
Light PA	0.020	-0.005	**0.073**	0.012	-0.011	**0.063**	0.078	0.064	0.100
Moderate PA	**0.181**	**0.157**	**0.185**	**0.182**	**0.161**	**0.179**	**0.196**	**0.176**	**0.189**
Vigorous PA	**0.269**	**0.249**	**0.241**	**0.276**	**0.260**	**0.238**	**0.247**	**0.220**	**0.229**
**Follow-up**		**N=1174**			**N=1022**			**N=152**	
Inactivity	**-0.073**	-0.054	**-0.087**	**-0.084**	**-0.068**	**-0.100**	0.010	0.027	0.009
Light PA	**0.064**	0.049	**0.073**	**0.069**	0.051	**0.087**	0.044	0.059	-0.009
Moderate PA	**0.211**	**0.196**	**0.184**	**0.220**	**0.204**	**0.200**	0.146	**0.161**	0.080
Vigorous PA	**0.299**	**0.294**	**0.230**	**0.312**	**0.310**	**0.238**	**0.191**	**0.187**	0.158

FEV_1 = forced expired volume in one second; FVC = forced vital capacity; MMEF = maximum mid-expiratory flow; PA = physical activity. Results are expressed as Spearman rank correlation coefficients. Significant ($p<0.05$) correlations are indicated in bold characters.

Supplementary table 4: Multivariable analysis of the associations between physical activity levels and spirometry results in absolute volumes, overall and stratified by smoking status, Pneumolaus baseline (2014-2017) and follow-up (2018-2021), Lausanne, Switzerland.

	**All participants**			**Never or former smokers**			**Current smokers**		
	**FEV_1**	**FVC**	**MMEF**	**FEV_1**	**FVC**	**MMEF**	**FEV_1**	**FVC**	**MMEF**
**Baseline**		**N=1910**			**N=1604**			**N=306**	
Inactivity	0.035	0.039	0.018	0.032	0.338	0.017	0.052	0.064	0.026
Light PA	**-0.129**	**-0.144**	**-0.063**	**-0.152**	**-0.166**	**-0.080**	-0.005	-0.019	0.026
Moderate PA	-0.016	-0.023	-0.007	-0.037	-0.044	-0.023	**0.115**	0.109	0.085
Vigorous PA	**0.105**	**0.101**	**0.074**	**0.092**	**0.091**	**0.055**	**0.186**	**0.164**	**0.204**
**Follow-up**		**N=1174**			**N=1022**			**N=152**	
Inactivity	0.030	0.041	0.011	0.029	0.042	0.001	0.060	0.071	0.070
Light PA	**-0.094**	**-0.097**	**-0.078**	**-0.109**	**-0.117**	**-0.073**	-0.025	0.010	-0.119
Moderate PA	-0.003	-0.004	-0.020	-0.018	-0.023	-0.022	0.080	0.115	-0.034
Vigorous PA	**0.122**	**0.111**	**0.101**	**0.119**	**0.108**	**0.098**	0.130	0.125	0.105

FEV_1 = forced expired volume in one second; FVC = forced vital capacity; MMEF = maximum mid-expiratory flow; PA = physical activity. Results are expressed as beta-standardized coefficients. Statistical analysis conducted using multivariable regression adjusting for age (continuous) and body mass index (continuous), and for smoking categories (never, former, current) when all participants were considered. Significant ($p<0.05$) associations are indicated in bold characters.

Supplementary table 5: Coefficients of determination between physical activity levels and spirometry results in absolute volumes, overall and stratified by smoking status, Pneumolaus baseline (2014-2017) and follow-up (2018-2021), Lausanne, Switzerland.

	**All participants**			**Never or former smokers**			**Current smokers**		
	**FEV_1**	**FVC**	**MMEF**	**FEV_1**	**FVC**	**MMEF**	**FEV_1**	**FVC**	**MMEF**
**Baseline**		**N=1910**			**N=1604**			**N=306**	
Inactivity	**0.3%**	0.2%	**0.5%**	**0.4%**	**0.3%**	0.6%	0.0%	0.0%	0.0%
Light PA	0.0%	0.0%	0.5%	0.0%	0.0%	0.4%	0.6%	0.4%	1.0%
Moderate PA	**3.3%**	**2.5%**	**3.4%**	**3.3%**	**2.6%**	**3.2%**	**3.8%**	**3.1%**	**3.6%**
Vigorous PA	**7.2%**	**6.2%**	**5.8%**	**7.6%**	**6.8%**	**5.7%**	**6.1%**	**4.8%**	**5.2%**
**Follow-up**		**N=1174**			**N=1022**			**N=152**	
Inactivity	**0.5%**	0.3%	**0.8%**	**0.7%**	**0.5%**	1.0%	0.0%	0.1%	0.0%
Light PA	**0.4%**	0.2%	**0.5%**	0.5%	0.3%	0.8%	0.2%	0.3%	0.0%
Moderate PA	**4.5%**	**3.8%**	**3.4%**	**4.8%**	**4.2%**	4.0%	2.1%	**2.6%**	**0.6%**
Vigorous PA	**8.9%**	**8.6%**	**5.3%**	**9.7%**	**9.6%**	5.7%	**3.6%**	**3.5%**	**2.5%**

FEV_1 = forced expired volume in one second; FVC = forced vital capacity; MMEF = maximum mid-expiratory flow; PA = physical activity. Results are expressed as percentage. Significant ($p<0.05$) associations are indicated in bold characters.
